# Annual acknowledgement of reviewers

**DOI:** 10.1186/s12860-015-0052-x

**Published:** 2015-03-21

**Authors:** Tim Sands

**Affiliations:** BioMed Central, Floor 6, 236 Gray’s Inn Road, London, WC1X 8HB UK

## Abstract

The editors of BMC Cell Biology would like to thank all our reviewers who have contributed to the journal in Volume 15 (2014).

**Byung-Yoon Ahn**

South Korea

**Gabriela Alexandru**

United Kingdom

**Matilde Alique**

Spain

**Nagabhushana Anathamurthy**

India

**Meir Aridor**

USA

**Simon Arthur**

United Kingdom

**Atsushi Asakura**

USA

**Safinur Atay**

USA

**Hannah Bader**

USA

**Maria Balda**

United Kingdom

**Tamas Balla**

USA

**Ora Bernard**

Australia

**Viviana Michelle Berthoud Barrandeguy**

USA

**Ramesh Bhonde**

India

**Karen Bieback**

Germany

**Françoise Botterel**

France

**Jeffrey Brodsky**

USA

**Dorly Buchi**

Brazil

**Anton Burakov**

Russia

**Robert Burgoyne**

United Kingdom

**Anil Cashikar**

USA

**Yang Chen**

China

**Yen-Hsu Chen**

Taiwan

**Xingguo Cheng**

USA

**Angela Clerk**

United Kingdom

**Emauele Cocucci**

USA

**Maria Lucia Correa-Giannella**

Brazil

**Jesmond Dalli**

USA

**Michael Danielsen**

Denmark

**Javid Dar**

India

**Viola Dengler**

USA

**Abby Dernburg**

USA

**Tobias Dick**

Germany

**Edward Draper**

USA

**Denis Dupré**

Canada

**Matthias Falk**

USA

**Mark Field**

United Kingdom

**Marie-Dominique Filippi**

USA

**So-Ichiro Fukada**

Japan

**Catarina Gadelha**

United Kingdom

**Gennaro Galasso**

Italy

**Minghui Gao**

USA

**Joel Goodman**

USA

**Mark Gorrell**

Australia

**Marcelo Gottschalk**

Canada

**Ivana Grivicich**

Brazil

**Jeff Hadwiger**

USA

**Thilo Hagen**

Singapore

**Sun Haiji**

China

**Ryota Hashimoto**

Japan

**Naohiro Hashimoto**

Japan

**Sudan He**

China

**Eva Hedlund**

Sweden

**Mary Herbert**

United Kingdom

**Jörg Höhfeld**

Germany

**Otmar Huber**

Germany

**Andrea Huwiler**

Switzerland

**Robert Insall**

United Kingdom

**Bill Janssen**

USA

**Marko Jovic**

USA

**Lukas Kapitein**

Netherlands

**Thomas Kelly**

USA

**Seong-Gon Kim**

South Korea

**Bernd Knoll**

Germany

**Joan Krepinsky**

Canada

**Erna Geessien Kroon**

Brazil

**Adam Kwiatkowski**

USA

**Hung-Wen Lai**

Taiwan

**Christian Lanctôt**

Czech Republic

**Fiona Lewis**

United Kingdom

**Marc Liesa**

USA

**Lijun Liu**

USA

**Yan Liu**

USA

**Pingsheng Liu**

China

**Wei Liu**

United Kingdom

**Wei Luan**

Australia

**Xianghang Luo**

China

**Vladimir Lupashin**

USA

**Andrew Macdonald**

United Kingdom

**Manuela Martins-Green**

USA

**Tetsuo Maruyama**

Japan

**Karl Matter**

United Kingdom

**Stuart Meiklejohn**

United Kingdom

**Lindolfo Meirelles**

Brazil

**Andreas Merdes**

France

**Jane Millward-Sadler**

United Kingdom

**Ravi Misra**

USA

**Jeffrey Moffitt**

USA

**Takayuki Morisaki**

Japan

**Giovanni Morrone**

Italy

**Christian Morsczeck**

Germany

**Karsten Münstedt**

Germany

**Toshio Nakatani**

Japan

**Aziza Nassar**

USA

**Robert Newman**

USA

**Tsutomu Nohno**

Japan

**Marie Stampe Ostenfeld**

Denmark

**Nor Hayati Othman**

Malaysia

**Nobuaki Ozeki**

Japan

**Benoit Palancade**

France

**Janet Paluh**

USA

**Sathya Pandi**

India

**Christine Parachoniak**

USA

**Evangelia Pardali**

Germany

**Irena Pastar**

USA

**George Plopper**

USA

**Ioannis Prassas**

Canada

**Asmah Rahmat**

Malaysia

**Dayanidhi Raman**

USA

**Narendrakumar Ramanan**

India

**Raina Ramnath**

United Kingdom

**Nalam Rao**

India

**R. K. Rao**

USA

**Peter Reiser**

USA

**Rafael Roesler**

Brazil

**Karin Romisch**

Germany

**Nathan Sandbo**

USA

**Milena Saqui-Salces**

USA

**Richard Schäfer**

Germany

**Eyal Schejter**

Israel

**Harry Scherthan**

Germany

**Michael Schumacher**

USA

**Changshun Shao**

China

**Puran Sijwali**

India

**David Soll**

USA

**Barbara Stecca**

Italy

**Christian Steinkühler**

Italy

**Anna-Lena Ström**

Sweden

**Russell Szmulewitz**

USA

**Hiroyuki Tamemoto**

Japan

**Raija Tammi**

Finland

**Masato Tamura**

Japan

**Sebastian Thams**

Sweden

**Sabrina Tosi**

United Kingdom

**Cesar Trigueros**

Spain

**Andrey Tsvetkov**

USA

**Jaap Van Buul**

Netherlands

**Francesc Ventura**

Spain

**Sonja Vermeren**

United Kingdom

**Dmitri Vezenov**

USA

**Jianxun Wang**

China

**Shihua Wang**

China

**David Warburton**

USA

**Ken Watanabe**

Japan

**Frances Weis-Garcia**

USA

**Mark Weiss**

USA

**Traci Wilgus**

USA

**Akira Yamaguchi**

Japan

**Hua Yang**

USA

**Wei Yuan Yang**

Taiwan

**Ming Yao**

China

**Liangzhu Yu**

China

**Takashi Yurube**

Japan

**Bojun Zhao**

China

**Jingde Zhu**

China

